# Thrombocytopenia Warrants Clinical Attention in Hospitalized Patients with Sarcoidosis

**DOI:** 10.3390/jcm15208170

**Published:** 2026-10-21

**Authors:** Guy Dumanis, Tamir Ofek, Adi Gubbay, Coral Tal, Ilana Moshkovich, Noam Klegar, Paz Agam Ohayon, Noa Tennenhaus, Gad Segal, Yigal Farkash, Daniel Bendersky, Moria Tzibin, Reut Kassif Lerner

**Affiliations:** 1 Education Authority, Chaim Sheba Medical Center, Ramat Gan 5262000, Israel; 2 Faculty of Healthcare and Medicine, Ariel University, Ariel 40700, Israel; 3 Faculty of Medicine, Nicosia University, P.O. Box 24005, Nicosia CY-1700, Cyprus; 4 Gray Faculty of Heath Science and Medicine, Tel-Aviv University, Tel-Aviv 69978, Israel; 5 Medicine Division, Ort Melton School of Science. Bat-Yam 5964712, Israel; 6 Department of Pediatric Intensive Care, Safra Children’s Hospital, Chaim Sheba Medical Center, Tel-Hashomer 5265601, Israel

**Keywords:** sarcoidosis, hypercalcemia, cytopenia, thrombocytopenia, hematologic malignancy, prognosis

## Abstract

**Background:** Long-term survival in sarcoidosis patients can be predicted by several clinical and laboratory parameters. Hypercalcemia serves as a predominant, negative prognostic factor. In the current study, we aimed to identify other patient characteristics influencing outcomes over a 10-year period. **Methods:** This was a retrospective analysis of hospitalized sarcoidosis patients in a large, tertiary medical center. **Results:** During a 10-year period between 2014 and 2024, data from 89 sarcoidosis patients was collected using MDClone data-mining software. Overall, 13 patients suffered from hypercalcemia (median, corrected for blood albumin: 10.3 mg/dL [9.9–10.5] vs. 9.5 [9.2–9.8], *p* < 0.001). Patients with hypercalcemia had higher rates of renal failure (30.8% vs. 3.9%, *p* = 0.008), lower platelet counts (median 153 K/mcl [113–170] vs. 230 [190.7–263.5], *p* < 0.001), lower hemoglobin concentrations (median 10.2 g/dL [10.0–11.3] vs. 12.5 [10.8–13.6], *p* < 0.01), higher urea blood levels (median 88 mg/dL [46–130] vs. 38.5 [30–47.2], *p* < 0.001), higher creatinine levels (median 1.48 mg/dL [1.31–1.6] vs. 0.9 [0.76–1.05], *p* < 0.001), and lower albumin (mean 3.07 g/dL ± 0.47 vs. 3.57 ± 0.56, *p* = 0.003). Hypercalcemia was associated with lower, post-discharge survival rates (median 4.8 years [2.6–7.3] vs. 7.9 [3.3–12.1], *p* = 0.045) and higher rates of 3-year, post-discharge mortality (30.8% vs. 7.9%, *p* = 0.036). Thrombocytopenia was present in six patients (6.7%); four (66.7%) died within three years, compared with six of 83 patients without thrombocytopenia (7.2%; *p* = 0.001). In univariate analysis, hypercalcemia and thrombocytopenia were both associated with a higher risk of mortality within 3 years (HR = 4.11, 95% CI 1.16–14.57, *p* = 0.029 and HR = 13.92, 95% CI 3.88–49.93, *p* < 0.001, respectively). However, in a multivariate analysis, only patient age and thrombocytopenia continued to be associated with a statistically significant higher risk of death (HR = 7.04, 95% CI 1.46–33.99, *p* = 0.015). Five of the six patients with thrombocytopenia (83.3%) were subsequently diagnosed with hematologic malignancy, months to years after thrombocytopenia was identified. **Conclusions:** Thrombocytopenia was associated with higher three-year mortality and may represent a clinical warning sign for occult or subsequently diagnosed hematologic malignancy in patients with sarcoidosis.

## 1. Background

### 1.1. Markers of Prognosis in Sarcoidosis Patients

Sarcoidosis is a systemic granulomatous disorder of unknown etiology [1]. Generally, sarcoidosis is considered a benign disease but of potentially devastating clinical results in some patients [2]. While the lungs and intrathoracic lymph nodes are involved in more than 90% of cases, sarcoidosis is a multisystem disorder that may affect nearly any organs, resulting in a broad spectrum of clinical manifestations [1]. Hypercalcemia has long been considered one of the metabolic characteristics of the sarcoidosis, arising, most probably, from the granulomatous component of disease, frequently endangering patient kidney functions [3,4,5]. Other than calcium metabolism, there are several markers potentially serving as prognostic factors for these patients: Zhou et al. found that serum angiotensin-converting enzyme (ACE) levels and soluble interleukin-2 receptor blood levels serve as biomarkers for advanced sarcoidosis, signified by the authors as sarcoid with multiple organ involvement and abnormal calcium metabolism [6].

### 1.2. Hypercalcemia in Sarcoidosis

Chronic hypercalcemia has been traditionally accepted as an adverse prognostic factor in patients with sarcoidosis [7]. While hypercalcemia has been shown to be associated with decreased rates of spontaneous disease resolution [5], a separate study found that calcium serum levels in patients with active disease were comparable to those with non-active sarcoidosis and that there was no association between calcium levels and decreased quality of life [8]. Hence, the prognostic role of hypercalcemia in sarcoidosis remains unclear, especially in the context of mortality which has not been widely explored.

### 1.3. Thrombocytopenia as Part of Sarcoidosis

Hematological abnormalities are relatively uncommon in sarcoidosis [9], with lymphopenia and anemia being the most frequently reported findings [10]. Isolated thrombocytopenia, defined as a platelet count below 100 × 10^9^/L, is a relatively rare extrapulmonary manifestation of sarcoidosis [1]. The first occurrence of thrombocytopenia in sarcoidosis was documented in 1938, and its estimated incidence in retrospective studies was reported to be only 1% to 2% [11,12].

Three main pathophysiological mechanisms have been described for thrombocytopenia in sarcoidosis: immune-mediated platelet destruction, splenomegaly leading to hypersplenism, and granulomatous infiltration of the bone marrow. Immune-mediated peripheral destruction is considered the most prevalent mechanism, potentially accounting for more than 80% of sarcoidosis-related thrombocytopenia cases [11]. This mechanism is supported by bone marrow findings that frequently show megakaryocyte proliferation, suggestive of an immune destructive process [10]. Splenomegaly is observed in approximately 10% of sarcoidosis patients and may contribute to platelet sequestration and destruction through hypersplenism [13]. Granulomatous infiltration of the bone marrow, although rare, is also a reported mechanism that may potentially reduce platelet production [14]. Interestingly, complex cases may exhibit evidence of all three pathophysiological mechanisms simultaneously [10].

Sarcoidosis-associated immune thrombocytopenia (ITP) is often characterized by a severe and acute onset [13]. In clinical series, median platelet counts at presentation were often severely low, reaching 11 × 10^9^/L in one study, with 85% of patients having counts below 30 × 10^9^/L [11]. Patients may present with significant bleeding symptoms such as petechiae, ecchymosis, and mucosal bleeding [9,12]. The chronological relationship between the conditions varies; while sarcoidosis often precedes ITP onset (median interval of 48 months), simultaneous onset or relapse of both conditions is also frequent, occurring in up to 60% of cases [11].

Due to the rarity of this association, there are no specific guidelines for the management of thrombocytopenia in sarcoidosis [13]. Therapeutic approaches typically follow guidelines established for primary ITP, utilizing corticosteroids and intravenous immunoglobulin (IVIg) as first-line agents [11,14]. While early reports described sarcoidosis-associated ITP as potentially life-threatening and associated with mortality rates up to 15% due to bleeding, modern therapeutic management generally yields a favorable response, with recent series reporting no related deaths or severe bleeding episodes [11].

Despite the generally good outcome for the ITP component, the underlying sarcoidosis disease course in these patients often appears chronic, requiring prolonged corticosteroid therapy for longer than 36 months in up to 65% of cases. Furthermore, hematological abnormalities are recognized as potential indicators of severe systemic inflammatory activity. This critical finding suggests that low platelet count serves as a marker for a more severe or persistently active disease phenotype. Given the acute severity, chronic nature of the associated sarcoidosis, and the independent prognostic significance of thrombocytopenia, a comprehensive evaluation of its contribution to the risk of mortality is crucial for optimizing long-term clinical management and monitoring in sarcoidosis patients [11].

### 1.4. Aim of the Current Study

Given that pulmonary assessment, such as the results of bronchoalveolar lavage, is not directly related to prognosis [2], there is a need to identify reliable prognostic markers that could improve clinical decision-making and disease management. The present study aims to investigate prognostic factors in sarcoidosis using data from a large tertiary medical center. Specifically, we re-evaluated the prognostic significance of metabolic and hematologic biomarkers in sarcoidosis patients over a long follow-up period.

## 2. Methods

### 2.1. Study Variables

We analyzed data from hospitalized sarcoidosis patients, including demographic variables (age at hospitalization, gender, smoking status, and weight), clinical background (relevant comorbidities and chronic medication use) and laboratory parameters collected at the time of hospitalization. Serum calcium levels were corrected for hypoalbuminemia using the following formula: “Corrected calcium (mg/dL)” = “Serum calcium” + 0.8 × (4 − “Serum albumin”). For patients with albumin levels below 4 g/dL, corrected calcium values were used. Hypercalcemia was defined as calcium levels, corrected or uncorrected, greater than 10.4 mg/dL. Thrombocytopenia was defined as a platelet count below 100 × 10^9^/L, and anemia as a hemoglobin concentration below 10 g/dL. This study’s primary clinical outcomes included three-year mortality rate following hospitalization and overall mortality after discharge.

The index event was hospitalization in a patient with a recorded diagnosis of sarcoidosis, rather than necessarily the initial diagnosis or a relapse. Available patients’ data recorded the year or date of diagnosis, medication history, organ involvement, and hematologic malignancies. Medication entries were summarized as documented histories. Organ categories were nonexclusive. Missing or unclear diagnosis date, treatment, and organ involvement entries were retained as unknown. For hematologic malignancy, patients without a documented diagnosis, including those with no information in that field, were classified as not having a hematologic malignancy. Overall categorical percentages used all 89 patients as the denominator; subgroup percentages used the corresponding subgroup size. Only explicit calendar years were used to summarize diagnosis timing.

Additional clinical follow-up information on hematologic malignancies diagnosed after thrombocytopenia was summarized descriptively. The diagnostic interval was available as months to years, without exact patient-level intervals for a time-to-malignancy analysis. These subsequent diagnoses were considered separately from the malignancy variable in the original descriptive tables and Cox model; no time-dependent malignancy analysis was performed.

### 2.2. Statistical Analysis

Patients were categorized separately according to the presence or absence of hypercalcemia (Table 1a) and thrombocytopenia (Table 1b). Categorical variables were compared between groups using the Chi-square test or Fisher’s exact test, as appropriate. Continuous variables were assessed for normality using the Shapiro–Wilk test. Normally distributed variables were compared using the independent-sample *t*-test, while non-normally distributed variables were compared using the Mann–Whitney U test.

Survival analyses were performed using Cox proportional hazard regression models to evaluate associations between study variables and mortality outcomes. Both univariable and multivariable models were constructed. Hazard ratios (HRs) with 95% confidence intervals (CIs) were reported for all Cox regression analyses. Kaplan–Meier survival curves were generated to illustrate overall survival according to the presence of thrombocytopenia, anemia, and hypercalcemia, and differences were compared using the log-rank test.

Univariable Cox models examined each predictor separately and yielded unadjusted associations with mortality. The multivariable model included hypercalcemia, age, malignancy, thrombocytopenia, and anemia simultaneously, yielding associations conditional on these other variables. The purpose of this model was to make an exploratory adjustment of the associations of interest, rather than the development of a clinical prediction tool. Hypercalcemia and thrombocytopenia represent the two principal markers being compared; age addresses age-related differences in mortality risk, malignancy addresses an alternative contributor to cytopenia and mortality, and anemia accounts for a coexisting hematologic abnormality that may reflect broader illness burden. Including these variables permits a clinically interpretable assessment of whether the thrombocytopenia association persists conditional on these measured factors. However, 10 deaths provide only approximately two events per fitted covariate. Clinical relevance does not eliminate the resulting risk of overfitting, sparse-data bias, and imprecise estimates. Adjusted results are therefore interpreted alongside the unadjusted findings and their 95% confidence intervals as exploratory associations requiring confirmation rather than evidence of a validated or stable independent prognostic effect.

Data were retrieved using MDClone’s ADAMS platform, a self-service query tool that provides comprehensive patient-level data of wide-ranging variables in a defined time frame around an index event (mdclone.com). All statistical analyses were performed using R software (version 4.4.2). A two-sided *p* value < 0.05 was considered statistically significant.

## 3. Results

### 3.1. Study Cohort

Following approval by the Institutional Review Board (IRB approval # 1713-25-SMC), patients’ electronic medical records (EMRs) were reviewed and relevant data were retrieved. Over a 10-year period, data from 89 hospitalized patients diagnosed with sarcoidosis were collected. Table 1a presents the baseline, demographic data of the entire cohort, background comorbidities, chronic medication use, laboratory parameters, and clinical outcomes, according to their calcium blood levels. Patients were categorized into two groups: those with hypercalcemia (13 patients with albumin-corrected calcium above 10.4 meq/L) and those with normal calcium levels (76 patients). Demographic characteristics (gender, age upon index hospitalization, smoking status and weight) were not different between the two calcium groups. In terms of comorbidities, the rates of ischemic heart disease, diabetes, heart failure, malignancy, dementia, hyper- and hypothyroidism and background anemia did not differ between the two study groups. Chronic Kidney Disease (CKD) was more prevalent in the hypercalcemia group (30.8% vs. 3.9%; *p* = 0.008). The chronic use of both angiotensin-converting enzyme inhibitors (ACE-Is) and thiazide diuretics did not significantly differ between the two study groups.

Regarding laboratory parameters, the hypercalcemia group showed markedly elevated levels of urea and creatinine (median 88 [46, 130] vs. 38.5 [30, 47.25], *p* < 0.001 and median 1.48 [1.31, 1.6] vs. 0.9 [0.76, 1.05], *p* < 0.001 respectively). Additionally, patients with hypercalcemia had lower concentrations (g/dL) of albumin (mean 3.07 ± 0.47 vs. 3.57 ± 0.56, *p* = 0.003), lower hemoglobin (g/dL, median 10.19 [10.03, 11.31] vs. 12.5 [10.79, 13.56], *p* = 0.005) and lower platelet counts (K/per mcl, median 153 [113, 170], vs. 230 [190.75, 263.5], *p* < 0.001). Liver function tests, lactate dehydrogenase (LDH) levels and white blood cell counts were not significantly different between the two study groups.

In terms of clinical outcomes, patients in the hypercalcemia group had significantly shorter median, post-discharge survival (4.8 years [2.6, 7.3] vs. 7.9 years [3.27, 12.1], *p* = 0.045). These patients also had a significantly higher rate of post-discharge, 3-year mortality (30.8% vs. 7.9%, *p* = 0.036).

Thrombocytopenia was present in six of 89 patients (6.7%), while 83 patients did not have thrombocytopenia (Table 1b). Four of the six patients with thrombocytopenia died within three years (66.7%), compared with six of 83 without thrombocytopenia (7.2%; *p* = 0.001). Mean age was 65.52 ± 17.71 versus 58.76 ± 15.41 years (*p* = 0.307). Hypercalcemia was present in three of six patients with thrombocytopenia (50.0%) versus 10 of 83 without thrombocytopenia (12.0%; *p* = 0.038), and malignancy was recorded in three of six (50.0%) versus six of 83 (7.2%; *p* = 0.013). Median hemoglobin was lower in the thrombocytopenia group (9.21 [IQR 8.30–11.13] versus 12.07 [10.59–13.31] g/dL; *p* = 0.031). The comparison of other demographic characteristics, comorbidities, chronic ACE-inhibitor and thiazide use, laboratory parameters, and survival time is presented in Table 1b.

Thrombocytopenia was defined as a platelet count <100 × 10^9^/L. Hypercalcemia was defined as serum calcium >10.4 mg/dL, with albumin correction when albumin was <4 g/dL. Values are n (%), mean (SD), or median [IQR], as indicated. The following abbreviations are used: ACE, angiotensin-converting enzyme; ALT, alanine aminotransferase; AST, aspartate aminotransferase; COPD, chronic obstructive pulmonary disease; CRP, C-reactive protein; IQR, interquartile range; LDH, lactate dehydrogenase; SD, standard deviation; WBCs, white blood cells.

The last row describes five subsequent hematologic malignancies among the six patients with thrombocytopenia, diagnosed months to years after thrombocytopenia was identified. It is separate from the original malignancy variable above. Dashes indicate that a comparable incidence analysis and *p*-value were not calculated. Subgroup-specific sarcoidosis treatment, splenic involvement, and bone marrow involvement were not available for this comparison.

As presented in Table 2, hypercalcemia, age, malignancy and thrombocytopenia were all associated with an increased hazard ratio for mortality within 3 years of admission. In a multivariate analysis, age and thrombocytopenia remained statistically associated with this clinical outcome in the exploratory model (HR = 1.06 [1.00, 1.11], *p* = 0.042 and HR = 7.04 [1.46, 33.99], *p* = 0.015, respectively).

**Figure 1:**
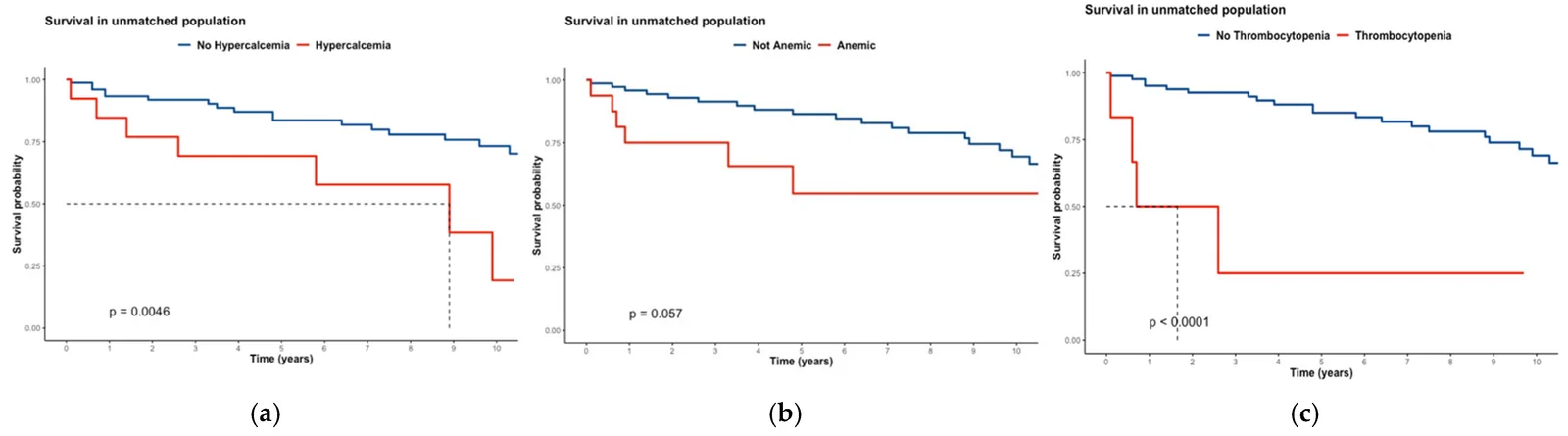
Kaplan–Meier curves for accumulated mortality. (**a**) According to hypercalcemia; (**b**) according to anemia; (**c**) according to thrombocytopenia.

Figure 1 presents the crude, accumulated mortality of sarcoidosis patients according to the existence of hypercalcemia (Figure 1a), anemia (Figure 1b) and thrombocytopenia (Figure 1c).

### 3.2. Additional Clinical Characterization

An explicit year of sarcoidosis diagnosis was available for 83 of 89 patients (93.3%): median: 2012 (IQR 2009–2017); range: 1977–2024. Two additional records described longstanding disease; four had no usable diagnosis date. Prednisone was recorded in 50 patients (56.2%), methotrexate in nine (10.1%), and azathioprine in four (4.5%). No treatment was recorded in 34 patients (38.2%), while treatment information was missing in two (2.2%). Hematologic malignancies were identified in the medical records of five patients (5.6% of the cohort). Updated clinical follow-up identified subsequent hematologic malignancy in five of the six patients with thrombocytopenia (83.3%), diagnosed months to years after thrombocytopenia was identified. The remaining 84 patients (94.4%), including those without information in the hematologic-malignancy field, were classified as not having a hematologic malignancy. The organ distribution and hematologic diagnoses are summarized in Table 3.

## 4. Discussion

Hypercalcemia is a known phenomenon associated with sarcoidosis, affecting approximately 6–11% of patients, with its pathophysiology thoroughly described in the literature [15]. Although initial presentation of hypercalcemia is rarely seen [16], its presence may indicate an acute disease phase and a high disease burden. Persistent hypercalcemia is considered one of the key indications for treatment initiation in sarcoidosis [5]. In addition, it was previously demonstrated that hypercalcemia serves as negative prognostic indicator in sarcoidosis, correlating with chronic-persistent disease, higher ACE levels, older age, hypergammaglobulinemia, and extrapulmonary involvement of organs such as the spleen, bone, and kidneys [17].

In this study, we aimed to establish whether hypercalcemia at the index hospitalization is associated with an increased mortality rate compared to normocalcemic patients, thus reinforcing its role as a prognostic factor in sarcoidosis.

In this study, 13 patients (14.6%) were identified with hypercalcemia. As shown in Table 1a, normocalcemic patients had a significantly longer median survival of 7.90 years, whereas patients with hypercalcemia demonstrated a significantly lower median survival of 4.80 years (*p* = 0.044). A Kaplan–Meier survival curve demonstrated a statistically significant difference in survival between the two groups (*p* = 0.0046). Moreover, 3-year mortality was significantly increased in the hypercalcemic group compared to the normocalcemic group (30.8% vs. 7.9%, *p* = 0.036). While 5-year mortality remained higher in the hypercalcemic group compared to the normocalcemic group (30.8% vs. 14.5%), the difference was not statistical significance (*p* = 0.221). This may suggest a short-term effect of hypercalcemia on survival, although over the long-term follow-up (of 5 years), it has no impact on survival.

These results should be taken into consideration when evaluating the role of hypercalcemia as a prognostic factor in sarcoidosis patients. In the multivariable analysis of three-year mortality, hypercalcemia was not statistically significant (HR 1.72, 95% CI 0.36–8.15; *p* = 0.493; Table 2). The reported five-year analysis was also not statistically significant (*p* = 0.254). However, this could be due to the small sample size. Larger cohort studies are needed to determine the role of hypercalcemia in the course of sarcoidosis. These findings highlight the importance of early recognition of hypercalcemia in sarcoidosis to optimize outcomes and reduce mortality risk. Sarcoidosis patients with hypercalcemia demonstrated significantly different clinical characteristics and laboratory parameters compared to those without hypercalcemia. Among all examined comorbidities (Table 1a), only renal failure demonstrated a statistically significant difference between groups (30.8% vs. 3.9%, *p* = 0.008), suggesting the consequential relationship between renal dysfunction and hypercalcemia, potentially mediated through dysregulation parathyroid hormone secretion leading to tertiary hyperparathyroidism. Accordingly, laboratory findings associated with severe renal impairment such as hyperphosphatemia (4.3 vs. 3.81 *p* < 0.001), elevated serum creatinine (1.48 vs. 0.9 *p* < 0.001), increased serum urea (88 vs. 38.5 *p* < 0.001), decreased hemoglobin (10.19 vs. 12.5 *p* = 0.005) and hypoalbuminemia (3.07 vs. 3.57 *p* = 0.003) were all statistically significant in the hypercalcemic cohort.

Thrombocytopenia was associated with higher three-year mortality in the univariable analysis (HR 13.92, 95% CI 3.88–49.93) and in the exploratory multivariable analysis (HR 7.04, 95% CI 1.46–33.99; *p* = 0.015). However, five of the six thrombocytopenic patients were subsequently diagnosed with hematologic malignancy. This finding shifts the interpretation from a potentially sarcoidosis-specific prognostic marker toward a possible warning sign of an underlying or evolving hematologic disorder. The wide confidence intervals and limited number of deaths warrant caution; differences in the magnitudes or statistical significance of the HRs for thrombocytopenia and hypercalcemia do not establish superior prognostic performance.

The five hematologic malignancies documented in the cohort comprised two cases of acute myeloid leukemia, two of diffuse large B-cell lymphoma, and one of myeloma. The subsequent diagnoses among five of six thrombocytopenic patients occurred months to years after thrombocytopenia was identified. This temporal sequence raises the possibility that thrombocytopenia preceded recognition of an occult malignancy in some patients; it does not establish that malignant disease was already present when the platelet count fell nor does it establish that sarcoidosis caused the malignancy. In a Spanish national registry analysis of deaths attributed to neoplasms, hematologic neoplasms accounted for a greater proportion of deaths among patients with sarcoidosis than among those without sarcoidosis [18]. That observation provides context for the clinical importance of hematologic malignancy but does not validate thrombocytopenia as a predictive marker.

Thrombocytopenia in sarcoidosis may have several explanations, including immune-mediated destruction, hypersplenism, marrow involvement, treatment effects, and hematologic malignancy. Splenic involvement was documented in five patients and bone marrow involvement in two, but these findings could not be linked to thrombocytopenia status. The available data therefore do not establish the mechanism of thrombocytopenia in individual patients. Our findings support considering occult or subsequently emerging hematologic malignancy when evaluating unexplained thrombocytopenia, rather than automatically attributing it to sarcoidosis. The observed proportion of 5/6 should not be interpreted as a generalizable probability of malignancy or as sufficient evidence for a standardized screening or biopsy strategy.

The principal limitations of this study are the retrospective, single-center design, the selected population of hospitalized patients, and the small number of thrombocytopenic patients and deaths. Five covariates were fitted to only 10 three-year deaths, making the adjusted estimates susceptible to overfitting and residual confounding. Although the sequence of thrombocytopenia followed by hematologic malignancy has been clarified, exact patient-level diagnostic intervals and the timing of malignancy relative to death were not available for this analysis. Subsequent hematologic malignancy was not evaluated as a time-varying exposure, and adjustment for the recorded malignancy variable does not establish that the thrombocytopenia association is independent of later hematologic disease. Disease stage, symptom duration, treatment timing and indication, and subgroup-specific splenic and bone marrow involvement remain incompletely characterized. This study does not establish a causal pathway linking sarcoidosis, thrombocytopenia, malignancy, and mortality, nor does it validate thrombocytopenia as a predictor of future malignancy. The findings are hypothesis-generating.

## 5. Conclusions

Thrombocytopenia in hospitalized patients with sarcoidosis was associated with higher three-year mortality. Five of six thrombocytopenic patients were subsequently diagnosed with hematologic malignancy over an interval of months to years, suggesting that unexplained thrombocytopenia may be a clinical red flag for an occult or subsequently recognized hematologic disorder. It should not be assumed to reflect sarcoidosis activity alone. These exploratory observations neither establish superiority over hypercalcemia as a prognostic marker nor demonstrate an independent predictive effect for future malignancy. Larger studies with confirmed diagnostic timing and detailed hematologic evaluation are needed before a specific surveillance strategy can be recommended.

## Figures and Tables

**Table 1:** (**a**) Study patients according to hypercalcemia status. (**b**) Study patients according to thrombocytopenia status.

(**a**)				
Variable	Whole cohort [*n* = 89]	Hypercalcemia *		*p* value
		No [*n* = 76]	Yes [*n* = 13]	
Calcium (mg/dL, median [IQR])	9.60 [9.20, 9.90]	9.50 [9.20, 9.83]	10.30 [9.90, 10.50]	<0.001
Demographic background				
Gender; M (N (%))	40 (44.9)	31 (40.8)	9 (69.2)	0.1
Age; years (mean (SD))	59.2 (15.6)	58.2 (15.4)	65.4 (15.9)	0.12
Smoking; N (%)	2 (2.2)	2 (2.6)	0 (0)	1
Weight; Kg (mean (SD))	75.9 (15.6)	75.8 (15.6)	77.0 (16.2)	0.82
Clinical background, N (%)				
IHD	14 (15.7)	12 (15.8)	2 (15.4)	1
Hypertension	33 (37.1)	28 (36.8)	5 (38.5)	1
Diabetes	27 (30.3)	21 (27.6)	6 (46.2)	0.2
Cardiac failure	2 (2.2)	1 (1.3)	1 (7.7)	0.27
Malignancy	9 (10.1)	7 (9.2)	2 (15.4)	0.61
Dementia	1 (1.1)	1 (1.3)	0 (0)	1
Hypo/hyperthyroid	4 (4.5)	4 (5.3)	0 (0)	1
Epilepsy	1 (1.1)	0 (0)	1 (7.7)	0.15
Renal failure	7 (7.9)	3 (3.9)	4 (30.8)	<0.01
Anemia	16 (18)	13 (17.1)	3 (23.1)	0.67
Chronic medication use, N (%)				
ACEi	14 (15.7)	13 (17.1)	1 (7.7)	0.68
Thiazides	5 (5.6)	4 (5.3)	1 (7.7)	0.55
Laboratory parameters				
Urea (mg/dL, median [IQR])	41 [32, 54]	38.50 [30, 47.25]	88 [46, 130]	<0.001
Creatinine (mg/dL, median [IQR])	0.95 [0.79, 1.20]	0.90 [0.76, 1.05]	1.48 [1.31, 1.60]	<0.001
ALT (IU/L, median [IQR])	21 [15, 37]	21 [14.75, 37.50]	21 [15, 31]	0.97
AST (IU/L, median [IQR])	24 [19, 34]	23 [18, 34]	26 [24, 32]	0.16
LDH (IU/L, median [IQR])	232 [189, 306]	223 [183, 292]	278 [248, 352]	0.065
Bilirubin (median [IQR])	0.60 [0.47, 0.85]	0.59 [0.47, 0.85]	0.65 [0.50, 0.76]	0.95
Albumin (g/dL, mean (SD))	3.50 (0.58)	3.57 (0.56)	3.07 (0.47)	0.003
WBCs; max (K/mcl, median [IQR])	8.8 [6.5, 12.5]	8.8 [6.4, 12.5]	8.8 [6.8, 10.9]	0.87
Platelets (K/mcl, median [IQR])	219 [174, 260]	230 [190.7, 263.5]	153 [113, 170]	<0.001
Hemoglobin (median [IQR])	11.85 [10.4, 13.2]	12.5 [10.8, 13.6]	10.2 [10.0, 11.3]	0.005
Clinical outcomes				
Median [IQR] Survival post admission; years	7.40 [3.10, 10.40]	7.90 [3.27, 12.10]	4.80 [2.60, 7.30]	0.045
Mortality 3 years post admission; N (%)	10 (11.2)	6 (7.9)	4 (30.8)	0.036
(**b**)				
Variable	Overall (*n* = 89)	Without thrombocytopenia (*n* = 83)	With thrombocytopenia (*n* = 6)	*p*-value
Platelets (×10^9^/L), median [IQR]	219 [174, 260]	226 [184.50, 261.50]	12 [3.75, 60]	<0.001
Hypercalcemia, n (%)	13 (14.6)	10 (12)	3 (50)	0.038
Male sex, n (%)	40 (44.9)	36 (43.4)	4 (66.7)	0.402
Age (years), mean (SD)	59.21 (15.56)	58.76 (15.41)	65.52 (17.71)	0.307
Smoking, n (%)	2 (2.2)	2 (2.4)	0 (0)	1.000
Weight (kg), mean (SD)	75.99 (15.58)	76.17 (15.53)	73 (18.49)	0.696
Ischemic heart disease, n (%)	14 (15.7)	14 (16.9)	0 (0)	0.584
Hypertension, n (%)	33 (37.1)	31 (37.3)	2 (33.3)	1.000
COPD, n (%)	7 (7.9)	6 (7.2)	1 (16.7)	0.397
Diabetes, n (%)	27 (30.3)	27 (32.5)	0 (0)	0.172
Heart failure, n (%)	2 (2.2)	1 (1.2)	1 (16.7)	0.131
Malignancy, n (%)	9 (10.1)	6 (7.2)	3 (50)	0.013
Dementia, n (%)	1 (1.1)	1 (1.2)	0 (0)	1.000
Hypo-/hyperthyroidism, n (%)	4 (4.5)	4 (4.8)	0 (0)	1.000
Epilepsy, n (%)	1 (1.1)	1 (1.2)	0 (0)	1.000
Renal failure, n (%)	7 (7.9)	5 (6)	2 (33.3)	0.069
ACE inhibitors, n (%)	14 (15.7)	14 (16.9)	0 (0)	0.584
Thiazides, n (%)	5 (5.6)	5 (6)	0 (0)	1.000
Urea (mg/dL), median [IQR]	41 [32, 54]	41 [32, 52.50]	53.50 [46, 79]	0.100
CRP, median [IQR]	27.76 [7.89, 87.55]	27.76 [8, 93.08]	26.28 [14.99, 42.95]	0.508
Creatinine (mg/dL), median [IQR]	0.95 [0.79, 1.20]	0.92 [0.78, 1.12]	1.25 [1.11, 1.31]	0.090
ALT (IU/L), median [IQR]	21 [15, 37]	21 [15, 35.50]	35 [13.25, 49.25]	0.706
AST (IU/L), median [IQR]	24 [19, 34]	24 [19, 32]	28 [17.75, 40.50]	0.768
LDH (IU/L), median [IQR]	232 [189, 306]	230 [188, 303]	281 [234, 343]	0.310
Bilirubin, median [IQR]	0.60 [0.47, 0.85]	0.59 [0.46, 0.84]	0.79 [0.66, 1.22]	0.066
Albumin (g/dL), mean (SD)	3.50 (0.58)	3.51 (0.58)	3.37 (0.58)	0.561
Maximum WBCs (×10^9^/L), median [IQR]	8.76 [6.53, 12.49]	8.92 [6.41, 12.54]	7.80 [7.01, 8.69]	0.793
Hemoglobin (g/dL), median [IQR]	11.85 [10.42, 13.26]	12.07 [10.59, 13.31]	9.21 [8.30, 11.13]	0.031
Survival from hospitalization (years), median [IQR]	7.40 [3.10, 10.40]	7.50 [3.40, 10.70]	1.50 [0.62, 2.53]	0.008
Three-year mortality, n (%)	10 (11.2)	6 (7.2)	4 (66.7)	0.001
Hematologic malignancy diagnosed after thrombocytopenia, n/N (%)	—	—	5/6 (83.3)	—

* Corrected Ca = measured Ca + 0.8 (4—measured albumin); if albumin < 4. IHD = ischemic heart disease, COPD = chronic obstructive pulmonary disease, ALT = alanine transaminase, AST = aspartate transaminase, LDH = lactate dehydrogenase, WBCs = white blood cells. Abbreviations: ACE = angiotensin-converting enzyme; ACE-I = ACE inhibitor; AKI = acute kidney injury; Ca = albumin-corrected serum calcium; CKD = chronic kidney disease; eGFR = estimated glomerular filtration rate; ICU = intensive-care unit; IQR = inter-quartile range; IV = intravenous; SD = standard deviation.

**Table 2:** Univariate and multivariate analysis of mortality within 3 years of discharge.

Variable	Univariate		Multivariate	
	HR [95%CI]	*p*-Value	HR [95%CI]	*p*-Value
Hypercalcemia	4.11 [1.16, 14.57]	0.029	1.72 [0.36, 8.15]	0.493
Age	1.07 [1.01, 1.13]	0.019	1.06 [1.00, 1.11]	0.042
Malignancy	7.34 [2.07, 26.09]	0.002	2.92 [0.72, 11.88]	0.135
Thrombocytopenia	13.92 [3.88, 49.93]	<0.001	7.04 [1.46, 33.99]	0.015
Anemia	3.35 [0.94, 11.87]	0.061	1.50 [0.31, 7.22]	0.615

Hypercalcemia, corrected for blood albumin > 10.4 meq/ml, thrombocytopenia < 100 K/mcl, anemia < 10 g/dL.

**Table 3:** Additional clinical patients’ characteristics.

Characteristic	n (%) of 89
Documented medication history	
Prednisone	50 (56.2)
Methotrexate	9 (10.1)
Azathioprine	4 (4.5)
Hydroxychloroquine	1 (1.1)
No treatment recorded	34 (38.2)
Treatment information missing	2 (2.2)
Documented organ involvement	
Pulmonary involvement	66 (74.2)
Lymph-node involvement	18 (20.2)
Cardiac involvement	9 (10.1)
Hepatic involvement	8 (9.0)
Neurologic involvement	8 (9.0)
Ocular involvement	8 (9.0)
Cutaneous involvement	7 (7.9)
Splenic involvement	5 (5.6)
Osseous involvement	4 (4.5)
Bone marrow involvement	2 (2.2)
Gastrointestinal involvement	2 (2.2)
Articular involvement	2 (2.2)
Renal involvement	1 (1.1)
Organ information missing or unclear	4 (4.5)
Documented hematologic malignancies	
Any hematologic malignancy	5 (5.6)
Acute myeloid leukemia	2 (2.2)
Diffuse large B-cell lymphoma	2 (2.2)
Myeloma	1 (1.1)
No hematologic malignancy	84 (94.4)

## Data Availability

Research data are available with the corresponding author under request and in line with the local IRB restrictions.
